# The effects of interrupting prolonged sitting with intermittent activity on appetite sensations and subsequent food intake in preadolescent children

**DOI:** 10.1371/journal.pone.0188986

**Published:** 2017-12-29

**Authors:** Tiwaloluwa A. Ajibewa, Molly P. O’Sullivan, Matthew R. Nagy, Shannon S. Block, Leah E. Robinson, Natalie Colabianchi, Rebecca E. Hasson

**Affiliations:** 1 University of Michigan School of Kinesiology, Ann Arbor, Michigan, United States of America; 2 University of Michigan Childhood Disparities Research Laboratory, Ann Arbor, Michigan, United States of America; 3 University of Michigan School of Public Health, Ann Arbor, Michigan, United States of America; Vanderbilt University, UNITED STATES

## Abstract

**Background:**

Short-term and long-term exposure to prolonged sitting is associated with excess food intake and weight gain in children. Interrupting prolonged sitting with low-intensity activity has been shown to not alter hunger, satiety, or food consumption in children, however it is unclear whether interrupting sitting with high-intensity activity will alter appetite regulation in children.

**Purpose:**

The purpose of this study was to examine the acute effects of interrupting prolonged sitting with intermittent activity performed at varying intensities on hunger, satiety, prospective food consumption (PFC), and food intake in preadolescent children.

**Methods:**

Thirty-nine children (ages 7–11 years, 54% female, 33% overweight/obese) completed four experimental conditions in random order: 8 hours of sitting interrupted with 20, 2-minute low-, moderate-, or high-intensity activity breaks or 20, 2-minute sedentary screen time breaks. Exercise intensity corresponded with 25%, 50% and 75% of heart rate reserve, respectively. Hunger, satiety, and PFC were assessed using the Visual Analog Scale, at five time points (pre- and post-breakfast, pre- and post-lunch, and pre-dinner) during each experimental condition. Dietary compensation was assessed as total caloric intake during a post-condition dinner standardized to provide 70% of estimated daily energy requirements.

**Results:**

There was a significant effect of time on hunger, satiety, and PFC throughout each condition day (p< 0.001). There were no differences across conditions for hunger (sedentary: 4.9±0.3 cm, low: 5.0±0.3 cm, moderate: 5.1±0.3 cm, high: 5.1±0.3 cm, p>0.05), satiety (sedentary: 4.7±0.3 cm, low: 4.4±0.3 cm, moderate: 4.6±0.3 cm, high: 4.2±0.3 cm, p>0.05), and PFC (sedentary: 4.9±0.3 cm, low: 4.7±0.3 cm, moderate: 4.9±0.3 cm, high: 5.0±0.3 cm, p>0.05). There were no significant differences in post-activity food intake across conditions (sedentary: 1071.9±53.6 kcals; low: 1092.6±43.4kcals; moderate: 996.2±54.6kcals; high: 1138.7±62.8kcals, p>0.05). However, there was a significant effect of condition on energy balance (sedentary: +61.4±65.9 kcals, low: +74.9±57.6 kcals, moderate: -58.3±62.8 kcals, high: -391.2±77.9 kcals; p<0.001). There were no significant effects of weight status on hunger, satiety, PFC, post-activity food intake, and mean energy balance across conditions (all p’s>0.05).

**Conclusions:**

Interrupting prolonged sitting with physical activity of any intensity does not alter appetite sensations and subsequent food consumption in children. These data suggest that interventions targeting prolonged sitting with high-intensity intermittent activity may be an effective strategy to increase physical activity energy expenditure without increasing food intake, allowing for a short-term energy deficit in both healthy weight and overweight/obese children. Future studies should examine the long-term effects of interrupting prolonged sitting with activity on food consumption and weight status in preadolescent children.

## Introduction

Short-term and long-term exposure to sedentary behaviors are associated with excess food intake and weight gain in children and youth [1]. In response to a single session of sedentary video game play followed by an *ad libitum* lunch, increased food intake was observed in a group of healthy weight adolescent males [2]. In a longitudinal study examining the associations between sedentary behavior and weight gain, greater time spent engaged in sedentary behaviors was associated with increased BMI in children ages 9–15 years [3]. This relationship persisted even after adjustments were made for moderate-to-vigorous physical activity, gender, race, maternal education, hours of sleep, and healthy eating index. Furthermore, increased sedentary screen time is associated with unfavorable health outcomes including increased weight gain and decreased fitness in a dose-response manner, due to the increased food intake associated with the behavior [4].

Earlier research has attempted to address the question of whether interrupting sedentary behaviors could influence dietary intake. For example, researchers have previously investigated the impact of interrupting prolonged sitting with low-intensity intermittent activity on subsequent food intake and physical activity levels [1]. They determined that the interruption of prolonged sitting with low-intensity physical activity did not alter subjective appetite sensations or subsequent food intake in children, compared with the sedentary condition. It is unclear whether higher intensity activity alters dietary behavior in children, thus examining the influence of changes in prescription or dose need to be made.

Data from the exercise literature suggest exercise intensity does, in fact play a key role in short-term post-exercise dietary behavior. In a prior study which examined 24-hour energy intake in 15 obese adolescents ages 12–15 years, researchers observed a 10% reduction in energy intake following a bout of high intensity cycling exercise (75% VO_2_max), in comparison to the low-intensity (40% VO_2_max) and sedentary conditions [5]. Similarly, the same group of investigators later examined changes in dietary behavior (energy intake and appetite sensations) in response to acute exercise (high-intensity cycling performed three times for 10 minutes at 75% VO_2_max) in both lean and obese adolescents. They found no significant differences in subjective appetite in the healthy weight or obese adolescents after a single exercise session, although a reduction in subsequent energy intake in the obese cohort was observed [6]. These findings suggest that weight status may also play a vital role in short-term post-activity dietary behavior. No study has, to our knowledge, examined the effect of interrupting prolonged sitting with activities of varying intensities in healthy weight and overweight/obese preadolescent children.

It is widely accepted that the excess energy intake in the U.S. is approximately 100 kcals/day for 90% of the population, meaning that relatively minor changes in energy intake and expenditure adding up to 100 kcal/day could reduce rapid weight gain in most people, including children [7]. If interrupting prolonged sitting with activity produces a short-term energy deficit, repeated exposures to intermittent activity may have important implication for pediatric weight management over time. Thus, the purpose of this study was to examine the effect of interrupting prolonged sitting with 20, 2-minute intermittent activity breaks of varying intensities (low-, moderate-, and high-intensity) on appetite sensations [hunger, satiety, and prospective food consumption (PFC)] and post-activity food consumption in a cohort of healthy weight and overweight/obese preadolescent children. Our primary hypothesis was that interrupting prolonged sitting with intermittent bouts of high-intensity activity will decrease appetite sensations as well as post-activity food intake in children compared to the sedentary screen-time condition. Given the influence of time of day on appetite sensations [8], we examined changes in appetite sensations throughout the day as an exploratory aim. We also examined changes in appetite sensations and food intake by weight status. Based on previous research [6], it was hypothesized that interrupting prolonged sitting with high-intensity activity breaks would be associated with a reduction in subsequent energy intake in the overweight/obese participants.

## Methods and procedures

### Participants

Thirty-nine healthy weight and overweight/obese children between the ages of 7–11 years were recruited from the greater Ann Arbor and Ypsilanti, Michigan areas to participate in the Active Class Space study. Active Class Space examined the acute effects of intermittent physical activity on a range of metabolic, behavioral, cognitive, and psychological outcomes in elementary school-aged children. Participants were excluded if: (i) they were taking medications or diagnosed with diseases that could influence dietary intake, exercise ability, body composition, or glucose metabolism; (ii) they were previously diagnosed with any major illness/health condition since birth; (iii) were diagnosed with clinical depression or any other mental health disorder that may have influenced mood, emotions, or stress perception; or (iv) had any diagnosed food allergies. After confirming eligibility, participants and their parents were provided with a full description of the study and signed an informed assent and consent document, respectively. The University of Michigan institutional review board approved this study.

### Study design

**Fig 1:**
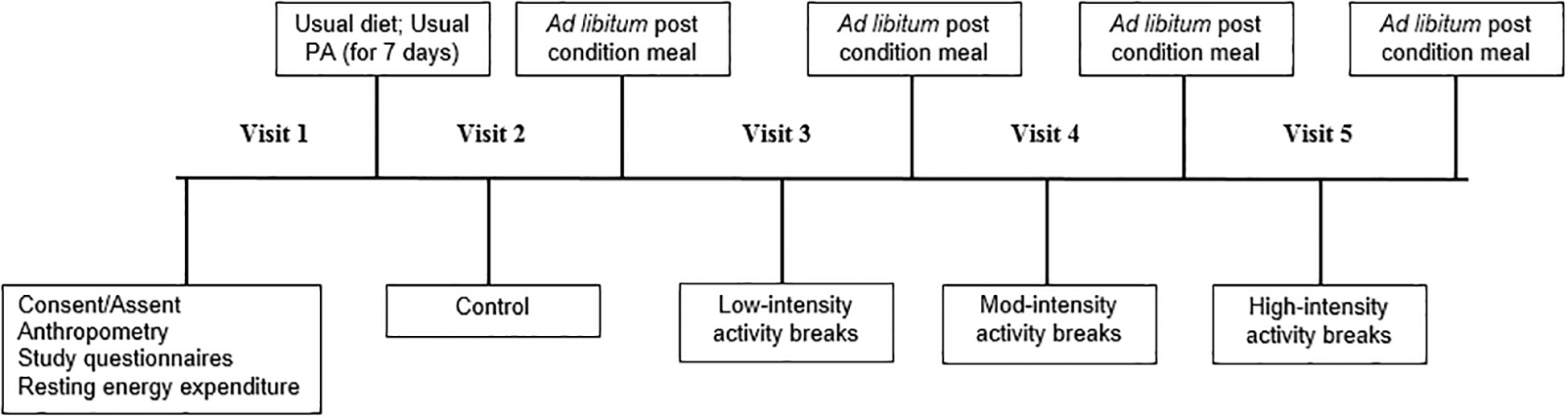
Overall study timeline. Visits 2–5 were completed in randomized order.

The timeline for each experimental condition is presented in Fig 1. Participants arrived at the Childhood Disparities Research Laboratory (CDRL) after an overnight fast where they completed anthropometric measurements, demographic questionnaires, and a measure of resting energy expenditure (REE). Prior to completing the experimental conditions, measures of usual physical activity were completed using accelerometry to accurately assess total daily energy requirements of each participant during their typical day to day activities in conjuction with measures of REE. Measurements are described below in the order in which they were performed.

### Visit 1 (pretesting visit)

#### Anthropometric measurements and demographic questionnaires

Body weight was measured using a digital scale (Doran Scales, Inc., Batavia, IL) to the nearest 0.1 kg, and barefoot standing height was measured to the nearest 0.1 cm using a wall-mounted stadiometer (ShorrBoard^®^, Weigh and Measure, LLC, Olney, MD). Both weight and height were measured using standardized procedures developed by Irwin Shorr [9]. Body mass index (BMI) and BMI percentiles were calculated using the CDC Body Mass Index Percentile Calculator for Children (CDC, 2000). Information collected regarding sex, age, and race/ethnicity were based on self-report.

#### Resting energy expenditure (REE)

REE was estimated using indirect calorimetry with a transparent ventilated metabolic hood system (ParvoMedics TrueMax 2400; Consentius Technology, Sandy, UT). Prior to each test, the calorimeter was calibrated with a reference gas mixture (95% O_2_, 5% CO_2_). A dilution pump was used to draw air from the ventilated hood at a rate of 15–30 L/minute for analysis. The day prior to measuring REE, participants were asked to avoid intense physical activity and to fast for at least 8 hours before arriving at the CDRL. The day of the test, participants arrived at the CDRL and were instructed to rest quietly for 15 minutes in supine position in a dimly lit, temperature controlled room. Oxygen consumption and carbon dioxide production were measured for 15–30 minutes, with steady state achieved when REE was stable over a period of 10 minutes. The final 5 minutes of this steady state data were used to assess REE.

#### Physical activity energy expenditure (PAEE)

Prior to the initiation of the experimental conditions, measures of habitual physical activity were assessed over a 7-day period for each participant using accelerometry (GT3X ActiLife, ActiGraph, Pensacola, FL). The devices were initialized to collect raw data at a frequency of 30 Hz. Raw accelerometer data were downloaded and integrated into 10s epochs using ActiLife software, version 6.11.8. A wear time of ≥600 minute/day and ≥4 days was used as the criteria for a valid day for baseline PAEE. Sleep time was manually entered using self-report logs, and marked as non-wear time. Intervals of ≥60 minutes of zero activity counts were also defined as non-wear time, allowing for up to 2 minutes of counts between 0 and 100 [10]. Non-wear times were excluded from the analyses. The Evenson Children 2008 cut-points were used to derive activity intensities (sedentary: 0 to 100 counts/minute; light physical activity: 101–2295 counts/minute; moderate: 2296–4011 counts/minute; and vigorous: 4012+ counts/minute) [11]. PAEE was calculated using the Williams Work-Energy 1998 algorithm [11]. The main outcome variable of interest was total daily PAEE. In addition to habitual PAEE, condition day PAEE was measured during each condition (8 hours in the laboratory) and the remainder of the condition day at home. These data regarding PAEE has been previously published [12].

#### Total daily energy requirements (TDEE)

Total daily energy requirements were calculated by estimating the sum of REE, average daily PAEE, and the thermic effect of food (approximately 10% of daily caloric requirements). Accelerometer-derived PAEE, has been previously shown to be positively correlated with TDEE measured by double labeled water in a sample of children [13].

### Experimental conditions (visit 2–5)

**Fig 2:**
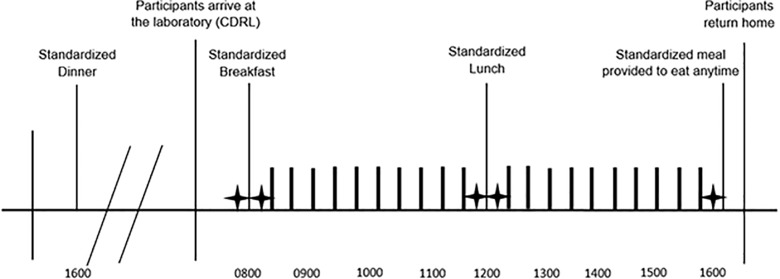
Timeline of condition day. Stars indicate when Visual Analog Scale was administered. Thick black bars indicate times when activity breaks were completed.

On four separate occasions, the participants returned to the CDRL to complete each experimental condition (visits 2–5), in random order. Participants completed four experimental conditions, which included 8 hours of sitting interrupted with 20, 2-minute low-, moderate-, or high-intensity activity breaks, or 20, 2-minute sedentary screen time breaks. Prolonged sitting was interrupted approximately every 18 minutes, with 10 breaks between breakfast and lunch, and another 10 breaks between lunch and the completion of the experimental condition, as seen in Fig 2. Exercise intensity for the low-, moderate-, and high-intensity conditions corresponded with 25%, 50%, and 75% of heart rate reserve, respectively. Heart rate reserve was estimated using the Karvonen formula [Maximum heart rate (220-age)–resting heart rate]. The Karvonen formula uses heart rate reserve to calculate exercise intensities based on the formula: Target Heart rate = (Heart Rate Reserve * Training %) [14]. Due to individual differences in resting heart rate, in part due to fitness level, a more accurate exercise intensity can be determined using heart rate reserve rather than estimating exercise intensity target from heart rate maximum alone. Heart rate reserve is a valid and reliable measure of exercise intensity in children [15, 16]. Heart rate was continuously monitored by the participants and study staff using heart rate monitors (Polar FT1 Heart Rate Monitor, Polar USA) to objectively guide the correct exercise intensity. All activities performed during the experimental conditions were pre-selected using the Compendium of Physical Activities for youth [17]. Activities for the low-intensity condition consisted of static stretching, standing, and yoga poses. Activities for the moderate-intensity condition consisted of push-ups, sit-ups, and age-appropriate calisthenics. Activities for the high-intensity condition consisted of vigorous calisthenics (e.g., jumping jacks). During the sedentary screen time condition, participants were given a tablet and instructed to select and play games for the two-minute period.

#### Randomization

Children completed each condition in random order. More specifically, for each condition, participants were randomized into different groups of two to three children completing the same condition, with the exception of one participant who completed one condition individually. Eleven participants started with the low-intensity condition first, another eleven participants started with the moderate-intensity condition, ten started the high-intensity condition, and seven started the sedentary screen time condition. Of the 24 possible condition orders (e.g., low-moderate-high-sedentary vs. sedentary-high-moderate-low) 18 were used in this study, with no more than 3 participants completing the experimental conditions in the same order. A maximum of five children were in the lab at one time. If different conditions were being completed on the same day, activity or sedentary screen time breaks were performed in separate areas of the laboratory with independent researchers guiding the activities. Throughout the remainder of the laboratory visit, participants engaged in a standardized set of common sedentary activities, which included watching videos, television programming, and playing board games. A minimum washout period of 4 days and a maximum period of two months was imposed between conditions.

### Standardized meals

Standardized meals were provided the evening prior to each experimental condition (~16:00 hours), at breakfast (08:00 hours), and at lunch (12:00 hours) of all condition days. Participants were asked to eat as much or as little of the standardized meals as they desired. If they did not complete the meal, the remaining food was collected and weighed to calculate dietary intake for that meal. All meals were constructed using a menu that was developed for children and youth by registered dieticians at the Nutrition Obesity Research Center’s Nutrition Assessment Laboratory at the University of Michigan. The specific contents of meals differed by individuals, as children could select from a standardized menu (S1 Table). Meals were however, standardized relative to total daily energy requirements (with the pre-condition dinner meal, breakfast, and lunch providing 40, 25, and 35% of estimated daily energy requirements, respectively). The proportions of kilocalories derived from carbohydrates, fats, and proteins were approximately 55, 30, and 15%, respectively, for each meal. An example of a standardized breakfast meal consisted of Cinnamon Toast Crunch^™^ with 2% milk, a Go-gurt^®^ tube, and a hard-boiled egg with salt and pepper. Over the course of each of the four experimental conditions, each participant received the same standardized meals and there were no differences in standardized meals (pre-condition dinner, breakfast, or lunch) consumed across conditions.

### Subjective appetite sensations

Before and immediately following each meal consumed in the laboratory, participants’ hunger, satiety, and PFC were assessed using the paper-based visual analog scale (VAS). Hunger was assessed with the question: “How strong is your desire to eat?” anchored by very weak (0 cm) and very strong (10 cm). Satiety was assessed using the question: “How full do you feel now?” anchored by not full at all (0 cm) and very full (10 cm). PFC was assessed with the question: “How much food do you think you could eat right now?” anchored by nothing at all (0 cm) and a large amount (10 cm). The participants were requested to put a vertical mark along the 10 cm horizontal line that best describes their appetite sensations at that moment, after which, the scales were collected by a member of the research staff. Previous studies have used VAS in laboratory settings to measure appetite sensations immediately following a meal [18, 1], the latter of which included the use of physical activity, comparable to this present study. VAS has been deemed highly reliable and valid in pediatric and adolescent populations [6].

### Dietary compensation meal (post-activity dinner meals)

Before leaving the CDRL (16:00 hours) on the day of the experimental condition, a standardized post-activity dinner meal was given to participants to be eaten at home. This meal was standardized to provide approximately 70% of estimated daily energy requirements, and the proportions of kilocalories derived from carbohydrates, fats, and proteins were approximately 55, 30, and 15%, respectively. Participants were instructed to eat as much or as little of the food as they desired and to return any uneaten food the following day. Returned food was weighed to calculate dietary intake and assess dietary compensation.

### Energy balance

Energy balance across conditions was calculated using the equation Energy balance = Total daily energy intake (breakfast kcals + lunch kcals + *ad libitum* dinner kcals)–Energy expenditure (REE +TEF+ condition day PAEE).

### Statistical analysis

All statistical analyses were performed using IBM SPSS Statistics 24. Results are expressed as means and standard error. Changes in appetite sensation (i.e., hunger, satiety, and PFC) were analyzed using a 2-way repeated measures analysis of variance (ANOVA), where the main effects of time (pre- and post- breakfast, pre- and post-lunch and pre-dinner) and condition (low-, moderate-, high-intensity and sedentary) were examined. Area under the curve or AUC [total AUC (0800-1600hrs), breakfast to lunch (0800–1200 hrs), and lunch to end-of-condition day (1200-1600hrs)] were also calculated using the trapezoidal method, for subjective appetite sensations. Changes in hunger, satiety, and PFC AUCs, as well as changes in dietary compensation (i.e., *ad libitum* post-activity food intake), and mean energy balance were analyzed using one-way repeated measures ANOVA where the main effects of condition were examined. BMI category (healthy weight, overweight/obese) was included as an interaction term in all analyses. If Mauchley’s test of sphericity was violated, data were corrected using Greenhouse Geisser epsilon (ε). When significant differences across experimental conditions and time were identified, post hoc pairwise comparisons with Bonferroni adjustments were conducted. An alpha level of p*<*0.05 was used for all analysis.

## Results

### Subject characteristics

Forty-four participants signed informed assents to participate in the study. Before data collection, four participants were unable to complete the study due to scheduling conflicts, and one participant was later deemed ineligible based on exclusion criteria. The remaining thirty-nine children (18 males, 21 females) ages 7–11 years, completed all four experimental conditions. From these 39 participants, 25 were healthy weight and 13 were overweight/obese. One of the participants (female, non-white, 10 years old) was classified as underweight based on the CDC growth charts but was included in the analysis as healthy weight for ease of interpretation. Average body mass index for the participants was 18.5±0.6 kg/m^2^, and 59% of the participants identified as non-white. A full description of the participant characteristics has been previously published [19]. The average REE and PAEE across participants was 1346.6 ± 46.9 and 247.8 ± 19.4 kcals/day for healthy weight children, and 1651.5 ± 70.7 and 385.3± 27.4 kcals/day for overweight/obese children. There were no significant differences across conditions of calories consumed from the standardized meals in both healthy weight and overweight/obese participants (all p’s>0.05; data not shown).

### Subjective appetite sensations

**Fig 3:**
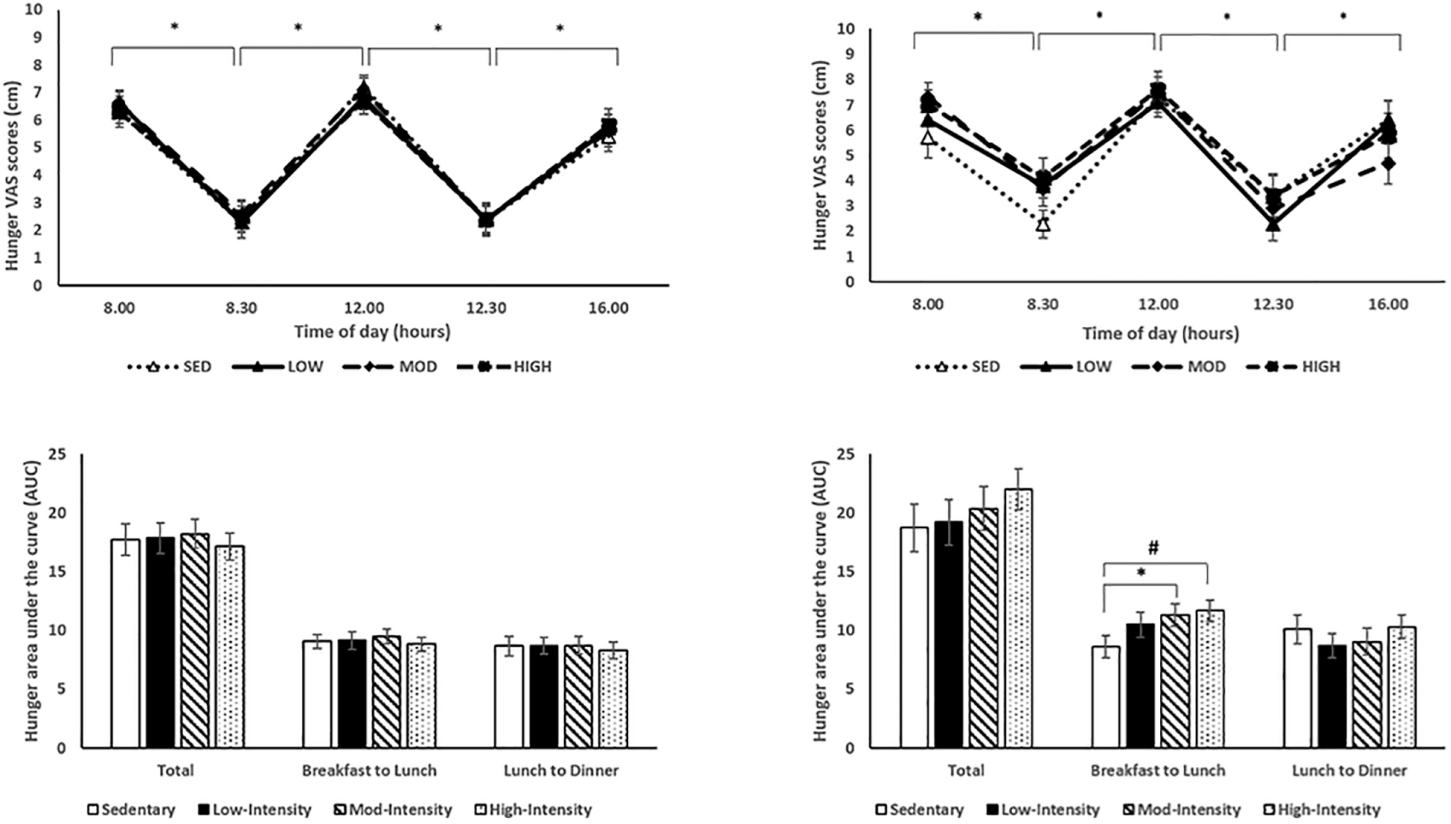
Subjective feelings of hunger throughout each experimental condition day. (A) Hunger appetite sensations throughout condition day. Asterisk denotes significant effect of time between pre- and post-meal times. (B) AUCs of hunger for each experimental condition. Normal weight participant data on the left, overweight/obese participant data on the right. Asterisk denotes significant effect of condition, # denotes trend at p = 0.09.

**Fig 4:**
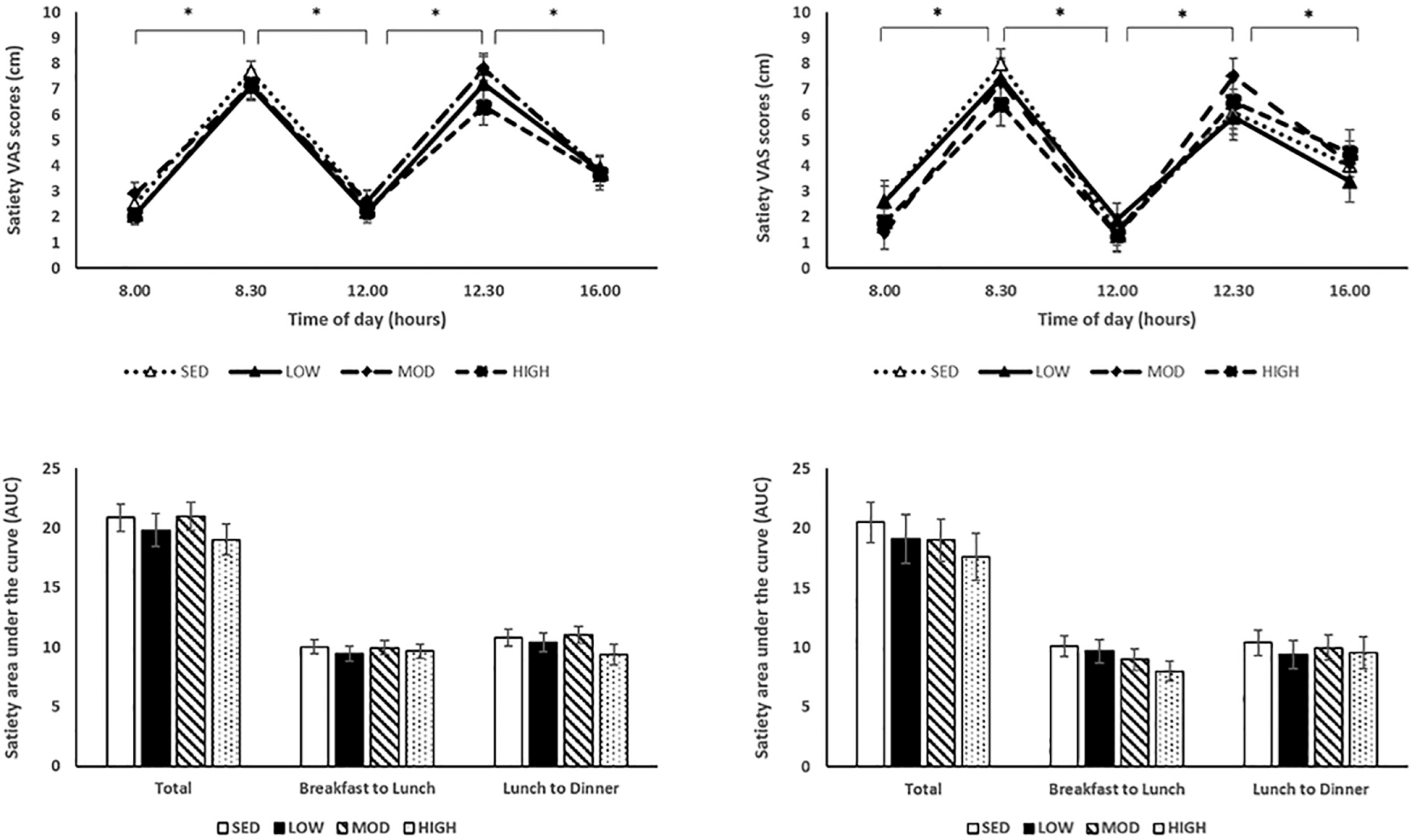
Subjective feelings of satiety throughout each experimental condition day. (A) Satiety appetite sensations throughout condition day. Asterisk denotes significant effect of time between pre- and post-meal times. (B) AUCs of satiety for each experimental condition. Normal weight participant data on the left, overweight/obese participant data on the right.

**Fig 5:**
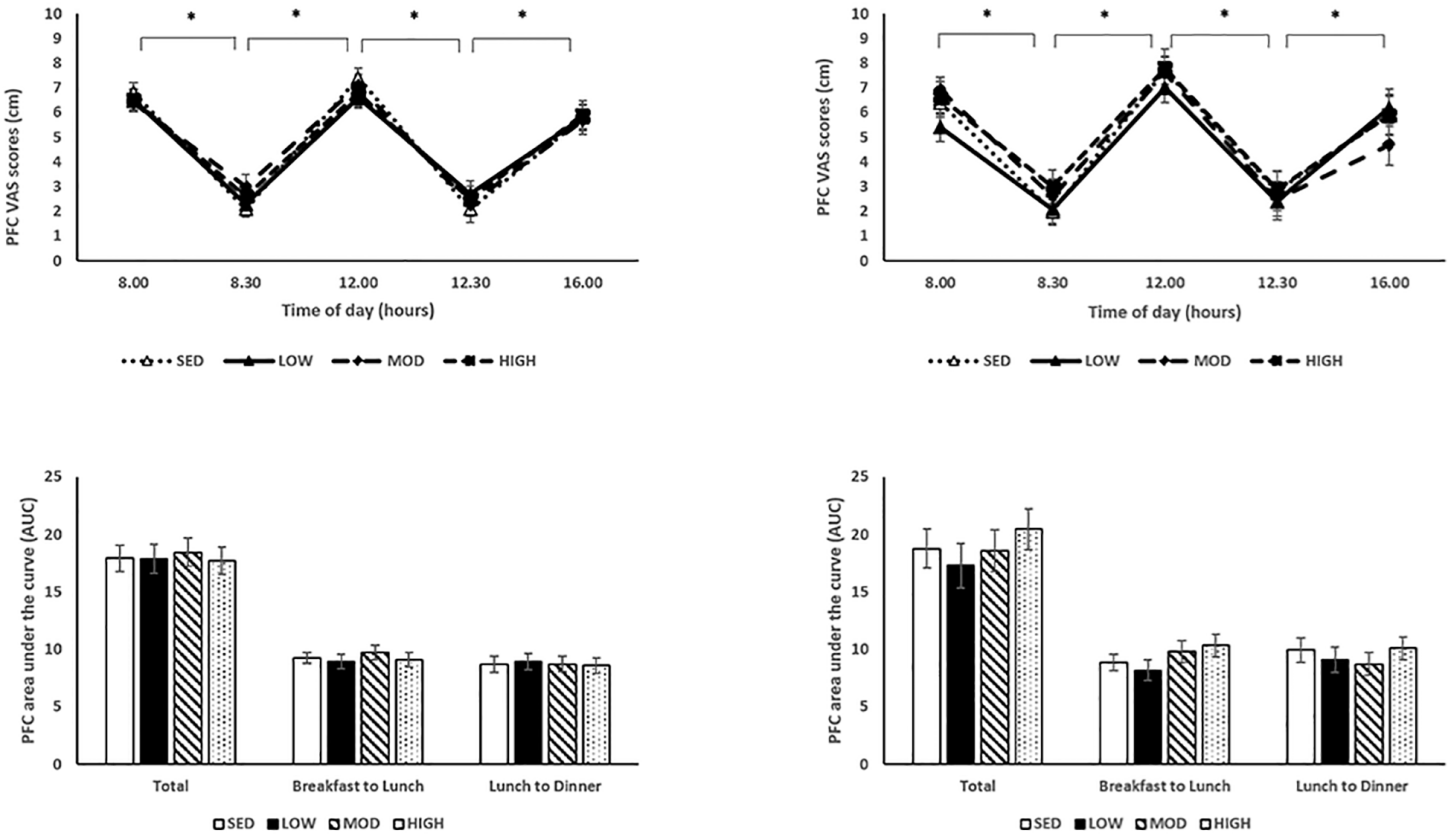
Subjective feelings of PFC throughout each experimental condition day. (A) PFC appetite sensations throughout condition day. Asterisk denotes significant effect of time between pre- and post-meal times. (B) AUCs of PFC for each experimental condition. Normal weight participant data on the left, overweight/obese participant data on the right.

Appetite sensations measured throughout each experimental condition for the normal weight and overweight/obese participants are presented in Figs 3A–5A. As expected, there were significant effects of time on hunger with significantly higher pre-meal hunger scores compared to post-meal scores (pre-breakfast: 6.5±0.3 cm vs. post-breakfast: 3.0±0.4 cm; pre-lunch: 7.2±0.3 cm vs. 2.7±0.4 cm; p<0.001; Fig 3A). There were significant effects of time on satiety with significantly lower pre-meal satiety scores compared to post-meal scores (pre-breakfast: 2.3±0.3 cm vs. post-breakfast: 7.3±0.4 cm; pre-lunch: 2.0±0.3 cm vs. post-lunch: 6.9±0.4 cm; p< 0.001; Fig 4A). Finally, there were significant effects of time on PFC with significantly higher pre-meal PFC scores compared to post-meal scores (pre-breakfast: 6.4±0.3 cm vs. post-breakfast: 2.5±0.3 cm; pre-lunch: 7.3±0.3 cm vs. post-lunch: 2.5±0.3 cm, p< 0.001; Fig 5A). There were no significant effects of condition on hunger, satiety and PFC (all p’s>0.05). There were no significant condition x time, condition x BMI, time x BMI, or condition x time x BMI interactions on hunger, satiety, and PFC (all p’s>0.05).

AUCs corresponding to each appetite sensation are presented in Figs 3B–5B. There were no differences in total AUC for each subjective appetite sensation across conditions (p>0.05). There was, however, a significant difference in breakfast to lunch hunger AUC across conditions (low-intensity: 9.8±0.7, moderate-intensity: 10.4±0.6, high-intensity: 10.3±0.6, sedentary: 8.8±0.6; p = 0.023), with a significantly lower breakfast to lunch AUC in the sedentary screen time condition compared to the moderate-intensity condition (p = 0.013). These findings were primarily driven by the overweight/obese children. A condition by BMI interaction was seen with generally higher breakfast to lunch hunger AUC with the overweight/obese children compared to the healthy weight children [(healthy weight: low-intensity: 9.147±0.7, moderate-intensity: 9.5±0.6, high-intensity: 8.8±0.6, sedentary: 9.1±0.6); (overweight/obese: low-intensity: 10.5±1.1, moderate-intensity: 11.3±0.9, high-intensity: 11.7±0.9, sedentary: 8.6±0.9; p = 0.028)].

There were no significant effects of condition on post-activity food intake with healthy weight children (low-intensity: 981.7±50.2 kcals, moderate-intensity: 926.1±63.0 kcals, high-intensity: 1016.3±72.5 kcals, sedentary: 999.0±61.9 kcals; p>0.05), nor with overweight/obese children (low-intensity: 1203.5±71.0 kcals, moderate-intensity: 1066.2±89.2 kcals, high-intensity: 1261.0±102.5 kcals, sedentary: 1144.9±87.5 kcals). There was no significant condition by BMI interaction on post-activity food intake (p>0.05).

**Fig 6:**
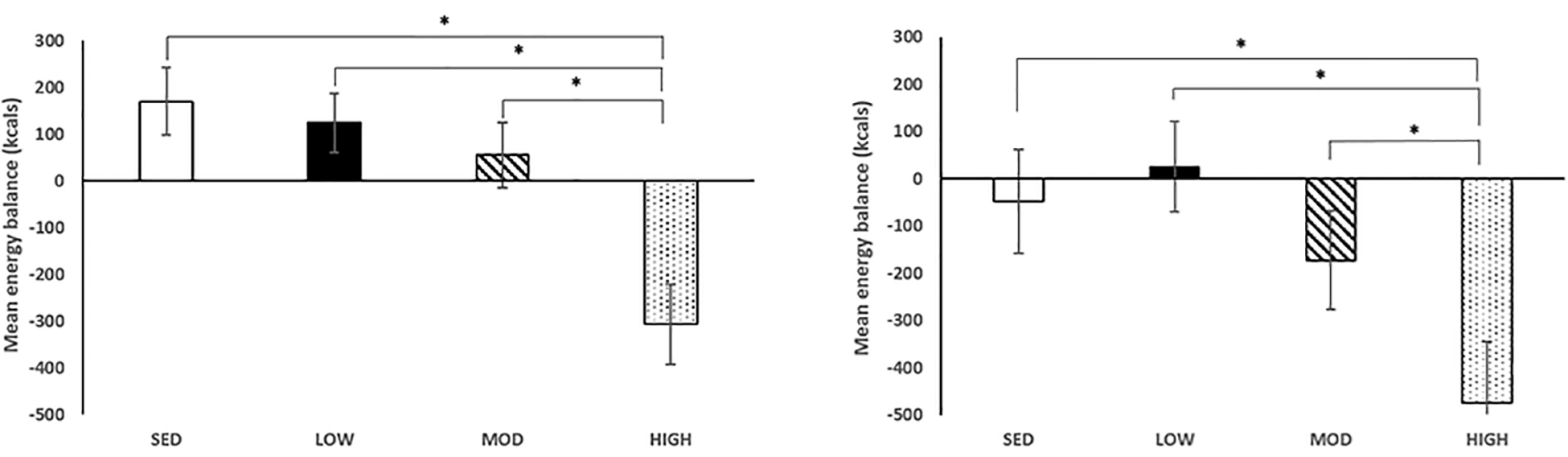
Mean energy balance across conditions. Asterisk denotes a significant difference between conditions. Normal weight participant data on the left, overweight/obese participant data on the right.

Mean energy balance change across conditions is presented in Fig 6. There was a significant effect of condition on energy balance across conditions with a significant energy deficit in the high-intensity condition compared to all other conditions in both the healthy weight (low-intensity: 124.4±63.7 kcals, moderate-intensity: 55.7±69.4 kcals, high-intensity: -306.8±86.1 kcals, sedentary: 170.7± 72.9 kcals; p<0.001) and overweight/obese participants (low-intensity: 25.4±96.0, moderate-intensity: -172.2±104.6, high-intensity: -475.6±129.9, sedentary: -48.0±109.9; p<0.001). There was no significant condition by BMI interaction (p>0.05).

## Discussion

The present study investigated the effects of interrupting prolonged sitting with varying intensities of physical activity on appetite sensations in a cohort of healthy weight and overweight/obese preadolescent children. Contrary to our hypothesis, interrupting prolonged sitting did not alter subjective feelings of hunger, satiety, PFC, or total AUC corresponding to appetite sensations across conditions. A significant difference in breakfast to lunch AUC in hunger was seen, however total AUC did not differ for hunger. Also, we did not observe differences in post-activity food consumption across conditions, illustrating that preadolescent children do not increase or decrease their food intake in response to interrupting prolonged sitting with low-, moderate-, or high-intensity activity. More importantly, high-intensity activity breaks elicited an energy deficit ranging between -307 kcals to -476 kcals in normal weight and overweight/obese children, respectively. These findings may have important implications on weight outcomes in preadolescent children over time, but additional research in this domain is warranted.

There have been several studies that have examined the influence of exercise on subsequent acute or chronic energy intake and appetite feelings [5]. With emerging evidence of the detrimental effects of sedentary behavior [20], along with the current increasing trends of inactivity, sedentary behavior, and increased screen time in children [20] novel strategies are needed to decrease sedentary behavior and excess food intake associated with this behavior in children. We previously demonstrated in the same group of participants that children did not decrease their physical activity away from the lab in response to interrupting prolonged sitting with low-, moderate- or high-intensity activity breaks [12]. In the case of high-intensity activity breaks, participants accumulated an additional 35 minutes of moderate-to-vigorous physical activity and expended an additional 150 kcals throughout the condition day as a result of activities performed in the laboratory. Taken together with the findings in the present study, both healthy weight and overweight/obese children do not appear to eat back the calories they burned during these activities, resulting in a -391 kcals and -58 kcals energy deficit during the high- and moderate-intensity condition days compared to an energy surplus of +61 kcals, +75 kcals during the sedentary, low-intensity condition days, respectively. In support of the belief that small changes in behavior can lead to changes in energy balance [7], it has been observed in children ages 2–7 years that a small yet chronic surplus in kilocalories can lead to an excess of 4.3 kg over a 10 year span [21], further highlighting the importance of our findings. If children can consistently increase their total daily PAEE and as a result, their 24-hour energy expenditure without a subsequent increase in caloric intake, the inclusion of intermittent activity breaks may have important implications for pediatric weight management programs. Future studies should examine the long-term effects of interrupting prolonged sitting with activity on appetite sensations, subsequent food intake and weight outcomes in children.

Our results examining the effects of interrupting prolonged sitting with physical activity on appetite sensations in children and adolescents are consistent with previous research [1]. Using three different experimental conditions (a day of uninterrupted sitting; a day of sitting interrupted with a 2-minute light-intensity walk break every 20 minutes; and a day of sitting interrupted with a 2-minute light-intensity walk break every 20 minutes as well as 2 × 20 minutes of moderate-intensity PA), these earlier investigations found that following an *ad libitum* buffet meal, children did not alter their food intake [1]. Our study adds to this literature by demonstrating neither healthy weight nor overweight/obese children eat back calories in response to interrupting prolonged sitting with moderate- or high-intensity activity breaks.

Our findings are inconsistent with data from the exergaming literature [22]. In a study that examined the effects of active gaming (via playing the Nintendo Wii^™^ Sports tennis game) on appetite and food intake in children ages 8–11 years, significant differences were observed across video gaming conditions. Participants in the seated gaming condition without access to food reported higher hunger, PFC, and lowered fullness scores compared with the two conditions wherein participants had access to food [22]. Although hunger AUC was higher from morning-to-lunch during the moderate-intensity condition, total AUC did not differ by condition day. Differences in study design and duration may explain the discrepancies between the exergaming findings and those of the present study. Rather than providing *ad libitum* food throughout the experimental conditions, participants in the present study were provided with standardized meals during the condition day and an *ad libitum* post-activity meal to be eaten at home. Also, children in the present study participated in a series of 2-minute activity breaks from 0800 hours until 1600 hours (8-hour condition day), rather than one continuous bout of active gaming that lasted 90 minutes. Additional research should determine the effects of interrupting prolonged sitting with intermittent vs continuous activity on appetite sensations and food intake in children.

An interesting finding that we observed was that participants consumed substantially more calories during the *ad libitum* post-activity meal compared to the pre-condition standardized dinner. Specifically, children consumed on average 623.7±29.8 kcals during the pre-condition meal compared to 1074.8±42.0 kcals during the *ad libitum* post-activity meal. The higher number of calories consumed was likely related to the larger portion size of the post-activity meal. Children were given 40% of their TDEE for the pre-condition meal whereas the post-activity meal consisted of 70% of TDEE. This is consistent with previous research in which greater energy intake was associated with larger portion sizes in preadolescent children [23]. Likewise, it has been hypothesized that people often choose and likely eat more when offered a larger unit size of food when given the option of more food [24]. Future research should address this study limitation by examining pre- and post-activity food consumption when given equal portion sizes.

There are many strengths and limitations of this study that should be noted. The racial/ethnic diversity of the study participants, experimental design, and the inclusion of standardized meals were strengths of this study. Data limitations included an inability to measure appetite hormones including leptin, active ghrelin, and Peptide YY (PYY) due to funding limitations. Such measurements would have enabled greater insight into the mechanistic role of appetite regulation within the current study. Additionally, we were unable to use gold standard measurements of TDEE [25] (i.e., doubly labeled water or direct calorimetry) to estimate daily caloric needs in the present study. However, the use of indirect calorimetry and accelerometry likely improved the accuracy of our caloric estimations compared to regression equations based on population data. Also, participants were not asked to complete the VAS at home following their *ad libitum* post-activity dinner because they were allowed to eat the compensatory meal throughout the night with no time restrictions. As such, we were unsure of the accuracy of the VAS at home compared to in the laboratory when they were given 20 minutes to consume their meal. If a child grazed throughout the night versus devoured their meal upon arriving at home, we would not be able to discern if the child was full because of the amount of food they consumed or the timeframe in which they finished the meal. Nevertheless, future studies should consider measuring VAS in the home environment. Finally, data collected in a laboratory setting cannot be readily generalized to other settings. Nevertheless, this well-controlled laboratory study gives additional insight into post-activity dietary compensation in children.

Overall, these results suggest interventions targeting prolonged sitting via the inclusion of intermittent activity, may be an effective strategy to increase physical activity energy expenditure without triggering increases in hunger and perceived food consumption while maintaining satiety and food intake in children. As schools are beginning to see the benefit of breaking up sedentary behavior through the implementation of activity breaks in the classroom, it is important to have found that these activity breaks do not impact food intake in children. Current literature on the matter is conflicting. Thus a better understanding of the relationship between exercise intensity, appetite sensation and post-activity food intake would be valuable in providing schools and teachers more information on how to adequately administer these breaks to best combat the childhood obesity.

## Supporting information

S1 Table Menu for standardized and post-condition meals. (DOCX)

S1 Dataset Table containing the raw data for all appetite sensations and food intake across conditions. (A) Raw VAS scores for all participants. (B) Raw food intake for all participants.
(XLSX)
