# Supplementary material for: The effects of interrupting prolonged sitting with intermittent activity on appetite sensations and subsequent food intake in preadolescent children
Source: PLoS One. 2017 Dec 29;12(12):e0188986. doi: 10.1371/journal.pone.0188986 (PMC5747427; doi:10.1371/journal.pone.0188986)
Supplement: S1 Table — (DOCX) [file pone.0188986.s001.docx]

| **Breakfast** | **Lunch** | **Dinner 1** | **Dinner 2** |
| --- | --- | --- | --- |
| Cereal with milk:   - Rice chex - Cinnamon Toast Crunch - Cheerios-plain - Frosted flakes   Toast:   - Wheat - White   Honey wheat bagel | Protein:   - Turkey - Ham - Roast beef - PB and Jelly - Hummus   Cheese:   - American - Cheddar - Swiss - None   Condiment:   - Ketchup - Mustard - Mayonnaise - None   Bread   - Wheat - White - Multigrain bun | 1. Penne pasta with marinara sauce  2. Penne pasta with meat sauce  3. Chicken nuggets  4. Grilled Cheese | 1. Penne pasta with marinara sauce  2. Penne pasta with meat sauce  3. Chicken nuggets  4. Grilled Cheese |
| **Sides** | **Fruits/Vegetables** | **Spreads/Dips** | **Beverages** |
| - Turkey bacon - Hardboiled egg - String Cheese - Greek Go-gurt (yogurt) - Strawberry yogurt - Almonds - Pretzels - Potato Chips - Tator tots - Rice - White dinner roll - Wheat dinner roll - White bread - Wheat bread - Popcorn - Granola bar - Cheese its | - Applesauce - Grapes - Seasonal fresh fruit - Bananas - Mandarin oranges - Cooked green beans - Corn - Cooked broccoli - Mixed green beans - Strawberries | - Peanut butter - Strawberry jam - Grape jelly - Hummus - American cheese - Swiss cheese - Cheddar cheese - Ketchup - Mustard - Mayonnaise | - Water - Apple juice - Orange juice - Skim milk - 2% milk - Whole Milk - Chocolate milk - Vanilla soy milk |
|  |  |  | **Desserts** |
|  |  |  | - Chocolate and vanilla pudding - Graham crackers - Chocolate chip cookies - Rice krispy treats |
